# Longitudinal Analysis of the Immunostimulatory Properties and Safety Profile of *Lacticaseibacillus rhamnosus* *LRa05* as a Dietary Supplement

**DOI:** 10.4014/jmb.2502.02053

**Published:** 2025-07-14

**Authors:** Jiayi Wu, Li Zhou, Sitong He, Xuna Tang, Jue Wang, Yanan Li

**Affiliations:** 1 School of Food Science and Pharmaceutical Engineering, Nanjing Normal University, Nanjing 210023, P.R. China; 2 Department of specialist clinic, Nanjing Stomatological Hospital, Medical School, Nanjing University 210008, P.R. China; 3 National Institutes for Food and Drug Control, National Key Laboratory for Quality Control of Pharmaceutical Excipients, Beijing, China; Shanghai Medical Device and Cosmetics Evaluation and Verification Center, Shanghai, P.R. China

**Keywords:** *Lacticaseibacillus rhamnosus* *LRa05*, immunostimulatory, safety profile, probiotic

## Abstract

Supplementation with appropriate doses of *Lacticaseibacillus rhamnosus* has been reported to potentially attenuate immune changes. While some progress has been made in studying the immune function of *L. rhamnosus* LRa05, a lack of systematic summaries has led to the neglect of its application in enhancing immune function. This study aimed to investigate the effects of *L. rhamnosus* LRa05 on the immune function of mice and to evaluate its safety profile as a dietary supplement. Mice were divided into three dosage groups (0.205, 0.410, and 1.230 g/kg body weight [BW]) and a negative control group (PBS), with each group orally gavaged for 30 consecutive days. The study assessed body weight, organ-to-body weight ratio, cellular immune function, humoral immune function, monocyte-macrophage phagocytic function and NK cell activity. Additionally, a subsequent 90-day subchronic oral toxicity study was conducted on rats to evaluate the safety of *LRa05* consumption. Significant differences were observed in the number of antibody-producing cells, carbon clearance function, and NK cell activity across all dosage groups compared to the negative control group, indicating that *L. rhamnosus* LRa05 has a superior ability to enhance immunity. During the 90-day subchronic oral toxicity experiment, no obvious damage was observed, demonstrating its good safety profile for consumption. This study underscores the immunopotentiating effects of *L. rhamnosus* LRa05 and its safety as a dietary supplement, recommending it as a promising candidate for integration into the food and health food industries.

## Introduction

Probiotics refer to living microorganisms that have beneficial effects on the host when ingested in sufficient amounts [1, 2]. Currently, the most studied are the genera *Lacticaseibacillus* and *Bifidobacterium* [3]. Clinical research and animal experiments have shown that reasonable intake of probiotics can treat and alleviate many diseases such as acute gastroenteritis [4], lactose intolerance [5], constipation [6] and colonic disorders, hepatitis [7, 8], alcoholic fatty liver [9, 10], obesity [11, 12], inflammatory bowel disease [13, 14], and diabetes [15, 16]. With further clearer understanding of their functionality and safety, probiotics would hold a larger market and greater potential as adjuvant therapy in biomedicine.

*L. rhamnosus* *LRa05*, a widely used probiotic, was characterized by a single circular chromosome spanning 2,989,571 base pairs (46.76% GC content), devoid of plasmid elements. Sequence analysis predicted 2770 protein-coding genes. ANI comparison with *L. rhamnosus* NCTC 13764 showed 97.52% identity, confirming its species-level assignment. Comprehensive safety assessments were conducted: no antibiotic resistance genes were detected via NCBI-AMR, CARD, or ResFinder, and no virulence or pathogenicity markers were found in VFDB or PathogenFinder analyses. These data collectively demonstrate the genomic safety of *LRa05*. Moreover, recent study highlighted that the minimum inhibitory concentrations (MICs) of various antibiotics against *LRa05* were equal to or less than the species-specific cut-off stipulated in the EFSA guidelines, indicating that *LRa05* remains susceptible to clinically relevant antibiotics, including ampicillin, gentamicin, kanamycin, streptomycin, erythromycin, clindamycin, tetracycline and chloramphenicol. However, *LRa05* is resistant to vancomycin, different from other *L. rhamnosus*, no plasmids or antimicrobial resistance genes were detected in *LRa05*, implying that its vancomycin resistance is an intrinsic factor. This phenotype may stem from the unique structural composition of its cell wall [17, 18]. Furthermore, unlike *L. rhamnosus* subspecies that do not ferment lactose, maltose or sucrose, *LRa05* produces large amounts of L-lactic acid. In addition, the results of *in vitro* experiments revealed that *LRa05* has strong tolerance to artificial gastric juice and bile salts and does not produce harmful metabolites or exhibit hemolytic activity. These genetic and functional characteristics demonstrate that *LRa05* has a good safety profile and lay the foundations for further studies on its efficacy in humans [19].

At present, research on the function of *LRa05* is primarily focused on weight loss, reduction of blood sugar levels, intestinal regulation, and other areas. Sun *et al*. used *LRa05* to intervene in obese mice. The weight gain of mice treated with *LRa05* slowed down, the fat cells in epididymal fat were smaller overall and lipid accumulation in the liver was reduced [20]. Wu *et al*. used *LRa05* to intervene in mice with type 2 diabetes. The fasting blood glucose and oral glucose tolerance of *LRa05* treated mice were significantly lower than those of model mice. Animal experiments have also shown that *LRa05* has gutlocalized immunomodulatory effects [21]. In addition, *LRa05* has been studied for its effects on gut flora in healthy adults, with results showing no significant changes in gut microbiota among individuals consuming *LRa05*, but an increase in the relative abundance of *Lactobacilli* and a decrease in *Sellimonas* abundance [22]. Several studies have shown that *L. rhamnosus* has the ability to modulate immunity [23-26]. *LRa05* could improve the production of specific cytokines, such as TNF-α, IL-10, and IL-1β, maintain Th1/Th2 balance, and control the intestinal inflammatory response in immunocompromised mice. Meanwhile, *LRa05* could promote SIgA production by regulating cytokines secretion, ensuring intestinal immune function [27]. Additionally, IL-1β and/or TNF-α participate in the upregulation of TLR2 mRNA levels in hepatocytes. The high level of TLR2 expression in the liver may have important implications for pathogen-host interactions or microbial signaling [28].

However, there are also clear evidence that *L. rhamnosus* may induce severe infections like endocarditis in a rat model and in human in particular cases [29, 30]. It is noted that there are few reports on the analysis of the enhanced systemic immune activity and long-term toxicity of this probiotic after prolonged use. This study aims to comprehensively investigate the effects of *L. rhamnosus* *LRa05* on the overall immune function of mice and its long-term safety for consumption. The study will include assessments of body weight, organ/body weight ratios, cellular immune function, humoral immune function, monocyte-macrophage function, and NK cell activity after 30 days of oral administration. Additionally, indicators from a 90-day oral toxicity test will be examined to provide theoretical support for the further development and application of *LRa05*.

## Materials and Methods

### Materials

Concanavalin A (ConA) and methylthiazolyltetr azolium (MTT) were purchased from Sigma Company; The YAC-1 mouse lymphoma cells (NK target cells) were purchased from Topai (China) Biotechnology Co., Ltd., RPMI-1640, supplemented with 100 U/ml benzylpenicillin, 100 μg/ml streptomycin, fetal bovine serum (FBS), Giemsa stain, Hank's solution (pH 7.2), SA buffer, PBS buffer, ink and erythrocyte lysis buffer were purchased from Solarbio Science & Technology Co., Ltd. (China); Sheep red blood cells (SRBC), chicken red blood cells and guinea pig serum (complement) were purchased from Molecular Toxicology Inc.; Iodonitrovinyl tetrazolium chloride (INT), Phenazine dimethyl sulfate (PMS) and Dimethyl sulfoxide (DMSO) were purchased from Sigma-Aldrich Corporation (America); Glacial acetic acid, Hydrochloric acid, Isopropanol, Glycerol, Agarose, Lithium lactate, Oxidized coenzyme I, Tris-HCl, Wenqi's solution, NP40 and Sodium carbonate were purchased from China National Pharmaceutical Group Corporation.

### The Preparation of *LRa05*

*L. rhamnosus* *LRa05* were purchased from the Wecare-bio.com, Ltd. (China). Batch Number: 23K0107W; Manufacturing Date: 17/10/2023; Expiration Date: 16/10/2025; Storage Conditions: The product should be stored at -18°C or below to maintain viability and efficacy. Format: The culture is provided in powder form, which ensures stability and ease of use in experimental settings. Humidity Control: The relative humidity during storage should be maintained below 50% to prevent moisture absorption, which can affect the quality of the culture. Container Specifications: The container for storing the bacterial powder should be made of materials with good sealing properties, moisture resistance, and oxygen resistance. Suitable materials include aluminum foil bags or sealed cans, which help in preserving the integrity of the culture. Colony counting was conducted before the animal experiments to adjust the quantity of bacteria to 2.0 × 10^9^ CFU/g.

### The Evaluation of Immune Function of *LRa05*

All experimental procedures were approved following the guidelines provided by the Animal Care and Animal Experiments of Nanjing Nomal University (NNU20200609). We determined the drug administration measurement according to the following principles: the recommended consumption content of *L. rhamnosus* *LRa05* is 0.2 g/d and 4.0 × 10^8^ CFU/g. The low, medium and high doses were set as 5 times, 10 times and 30 times of the recommended human consumption. Based on the adult body weight (60 kg), the body surface coefficient was 12.3, namely 0.205, 0.410 and 1.230 g/kg BW. Deionized water was set as the negative control group. *LRa05* of 0.205, 0.410 and 1.230 g were respectively made up to 20 ml with deionized water and administered by gavage at a dose of 20 ml/kg BW. Eight-week-old male BALB/c mice were supplied by SPF Biotechnology Co., Ltd., (China). BALB/c mice were randomly divided into 4 groups (48 mice per group) and grouped according to the groups in the Table S1. Forty-eight mice in each group were divided into four groups at random (12 mice per group): (1) NC group, orally administered with 0.2 ml deionized water by gavage; (2) Low dose group, orally administered with 0.205 g/kg BW *LRa05* by gavage; (3) Medium dose group, orally administered with 0.410 g/kg BW *LRa05* by gavage; (4) High dose group, orally administered with 1.230 g/kg BW *LRa05* by gavage. *LRa05* were dissolved in deionized saline, and the NC group was exclusively administered 0.2 ml of deionized saline. All the mice used in the experiment are conventional raised a week in the standard animal room (Temperature: 23 ± 2°C, Relative humidity: 40%-70%). The following immune indicators was determined after 30-day treatment of *LRa05*.

### Body Weight and Organ Index

The body weight of mice was measured and recorded daily. After the gavage experiment, the mice were killed by cervical dislocation, the thymus and spleen were quickly removed, the surrounding fascia was removed, and the blood on the surface of the organs was aspirated with filter paper, and then weighed. The organ to body weight ratio were calculated as follows:

$$\text{organ index (mg/g) =}\frac{\text{organ weight (mg)}}{\text{body weight (g)}}$$ (1)

### Delayed Type Hypersensitivity (DTH) - Sensitization Red Blood Cell (SRBC) Induced Mouse Plantar Thickening Method

SRBC-induced DTH reaction was performed with slight modification from the method described by Shivaprasad *et al*. [31, 32]. The mice in different groups, which had been administered treatment for 30 days, were sensitized on the left hind paw with 200 μl of 2% (v/v) SRBC on the 0th day. At 4 days after sensitization, the thickness of the left hind paw of the mice was measured, and the measurement site was marked. Then the left hind paw were challenged with 20 μl of SRBC to initiate the antigenic challenge. Paw thickness was measured using a vernier caliper at 0 hours and 24 h post-challenge. The difference in thickness of the mouse plantar swelling before and after the challenge was recorded as DTH.

### Proliferation Ability of Spleen Lymphocytes

Spleen lymphocytes proliferation was evaluated by MTT assay. After 30-day treatments, the spleens were harvested and spleen lymphocytes suspension was adjusted to 2 × 10^7^ cells per mL. After 200 μl splenocytes were added to each well, then add 800 μl RPMI 1640 culture separately to make the total volume 1 ml per well. Then 75 μl of Concanavalin A (ConA) (7.5 μg/ml) were added, and blank group with 75 μl medium. The cells were then cultured for 72 h in an incubator at 37°C with 5% (v/v) CO_2_. 4 h before the end of the culture, 0.7 ml of the supernatant was absorbed from each well, and 0.7 ml of the RPMI 1640 culture without calf serum was added. Subsequently, 50 μl of MTT solution was added for an additional 4 h of culture. Finally, 1 ml of acidic isopropanol was added to completely dissolve the purple crystals, and the optical density (OD) was measured at 570 nm (OD570). The proliferative capacity of lymphocytes was expressed by subtracting the optical density value with the ConA wells from the optical density values without the ConA wells.

### Detection of Antibody-Generating Cell-Jerne Modified Slide Method

After defibrillation treatment, 0.2 ml of a 2% (v/v) fresh sheep blood cell suspension was injected into the abdominal cavity of each mouse for 4 days. Subsequently, the spleens were aseptically harvested to prepare a cell suspension, fixed volume at 8 ml Hank's solution. The surface culture medium was mixed with an equal volume of Hank’s solution, separate into small test tubes, 0.5 ml of each tube, followed by the addition of 50 μl 10% SRBC and 20 μl of the spleen cell suspension. The mixture was then quickly homogenized and poured onto a glass slide coated with a thin layer of agarose. After solidification, the slide was incubated for 1.5 h in an incubator at 37°C with 5% (v/v) CO_2_. Then the antibody diluted with SA buffer (1:8) was added into the groove of the slide holder for 1.5-h incubating, after which the number of hemolytic plaques was counted.

### Determination of Half Hemolytic Value (HC_50_) of Serum Hemolysin

After defibrillation treatment, 0.2 ml of a 2% (v/v) fresh sheep blood cell suspension was injected into the abdominal cavity of each mouse for 4 days. Then the eyes were removed from mice immunized for 4 days, 1.5 ml of blood was removed and allowed to stand for 1 h, centrifugation (2,000 r/min, 4 min), the serum was collected, diluted in 300 × SA buffer, taking 0.1 ml of diluted serum in 96-well plates, then 0.05 ml 10% SRBC and 0.1 ml complement sequentially were added. Keep it in a constant temperature water bath at 37°C for 30 min, and terminate the reaction in an ice bath. Centrifugation (2,000 r/min, 10 min), then take supernatant and add Wenqi's solution, and set half of the hemolytic wells. Take 10% 0.0125 ml of SRBC and add Wenqi's solution to 0.2 ml, mix well, and let it stand for 10 min. The optical density (OD) of each well was measured on the microplate reader reader at 540 nm (OD_540_). The amount of hemolysin is expressed as half of the hemolysis value (HC_50_) and calculated using the following formula:

$${\text{Sample HC}}_{50}=\frac{\text{sample absorance value}}{\text{SRBC half hemolysis sample absorance value}}\times \text{diluting folds of serum}$$ (2)

### Carbon Clearance Assay

The method followed is a modification of the method described by Gonda, Tomoda, Shimizu, and Kanari (1990) [33]. Mice were given diluted Indian ink intravenously, when the ink is injected, time it out immediately. Blood was collected from retro-orbital plexus at an interval of 2 (t_1_) and 10 (t_2_) min. Add above blood (20 μl) into 2 ml 0.1% Na_2_CO_3_. Then the optical density (OD) of each well was measured on the microplate reader reader at 600 nm (OD_600_), using a 0.1% Na_2_CO_3_ solution as a blank control. In addition, mice were sacrificed, liver and spleen were removed, dried the viscera surface with filter paper and weighed separately. The weight of mice, liver and spleen were recorded separately. Calculate the phagocytic index according to formula:

$$k=\frac{\mathrm{lg}OD_{1}-\mathrm{lg}OD_{2}}{t_{2}-t_{1}}$$ (3)

$$\text{phagocytic index }\kappa =\frac{\text{body weight}}{\text{liver weight+spleen weight}}\times \sqrt[{3}]{k}$$ (4)

### Determination on Macrophage Phagocytosis Function in Mice

Each mouse was intraperitoneally injected with a 5% compressed suspension of chicken red blood cells. After an interval of 2.5 h, the mice were euthanized by cervical dislocation. The abdominal skin was incised in the middle and physiological saline was instilled into the abdominal cavity. The abdomen was gently rotated for 1 min to ensure thorough lavage. Subsequently, the lavage solution was aspirated and evenly distributed onto two glass slides, which were then transferred to a 37°C incubator for a 30-min incubation period. Following incubation, non-adherent cells were removed by rinsing with physiological saline and air-dried. Fixation was performed using a solution consisting of acetone and methanol in a ratio of 1:1, followed by staining with a 4% (v/v) Giemsa phosphate buffer for 3 min. The slides were then rinsed and dried with distilled water before calculating the total number of macrophages and chicken red cells within 100 macrophages. Phagocytosis percentage and index were calculated using the following formula:

$$\text{Percent of phagocytosis(\%)}=\frac{\text{number of macrophages having swallowed CRBC}}{\text{total macrophages}}\times 100\%$$ (5)

$$\text{phagocytosis index}=\frac{\text{CRBC having been swallowed by macrophages}}{\text{total macrophages having swallowed CRBC}}$$ (6)

### NK Cell Activity Assay

The LDH release assay was employed to determine NK cell activity. The effector cells were prepared by resuspending the spleen lymphocytes suspension (2 × 10^7^ cells/ml). The cell concentration of the target cells suspension was adjusted to 4 × 10^5^ cells/ml. Respectively, in RPMI 1640 complete culture medium containing 10%calf serumand. The effector-to-target (E:T) ratios of 50:1 were used for co-culturing the effector and target cells in a 96-well plate. The target cell natural release pore plus target cell and 100 μl of culture medium, the target cell maximum release pore plus target cell and 1% NP40 m 100 μl, followed by incubation at 37°C for 4 h. After incubation, 100 μl of the supernatant was collected and mixed with 100 μl LDH matrix solution, which reacted in a dark place for 10 min. 30 μl HCl (1 mol/l) was then added to each well, and absorbance at 490 nm was measured using a microplate reader. NK cell activity was calculated using the following formula:

$$\text{NK cell activity(\%) =}\frac{\text{OD in experimental release-OD in spontaneous release}}{\text{OD in maximal release-OD in spontaneous release}}\times 100\%$$ (7)

### Subchronic Oral Toxicity

Weaned rats were supplied by Hangzhou Medical College (20211019Aazz0100018408, and the rats were randomly divided into 4 experimental groups (*n* = 20, 10 males and 10 females). (1) NC group, orally administered with 0.2 ml deionized water by gavage; (2) Low dose group, orally administered with 2.05 g/kg BW *LRa05* by gavage; (3) Medium dose group, orally administered with 4.10 g/kg BW *LRa05* by gavage; (4) High dose group, orally administered with 8.20 g/kg BW *LRa05* by gavage. Record weight weekly and calculate food utilization rate. After 90-day treatment, the following indicators were recorded. The brain, heart, liver, spleen, kidney, testes/ovaries, epididymis/uterus, adrenal gland and thymus of each rat were weighed and the organ-to-body weight ratio (organ weight/fasting weight before execution) was calculated. Histopathological examination was performed on the tissues of experimental rats. Simultaneously, hematological analysis, blood coagulation time measurement, blood biochemistry testing for electrolytes and urine routine were conducted.

### Design Dose Conversion

The 90-day oral toxicity dose (low, medium, and high) of 2.05, 4.10, 8.20 g/kg BW subjects were mixed into the diet at 8% of the body weight. After 90 d, the actual dose was calculated according to the average body weight and daily food intake (Table 1). The actual dose was calculated using the following formula:

$$\text{The actual dose}=\frac{\text{Daily intake}\times \text{Design dose}}{\text{middle weight}\times 8\%}$$ (8)

### Statistical Analysis

All experimental data are presented as mean ± standard deviation (x ± s) and compared with the negative control group. Use Excel and Graphpad Prism 8.0 software for data processing and statistical analysis. The measurement data were analyzed by one-way anova, or two independent samples *t*-test. If the variance was uneven, progressiveness variables were converted. If the variance was still uneven, the rank sum test was used instead. Rank sum test was used to analyze the hierarchical data.

## Results

### Immune Function Experiments

**Fig. 1:**
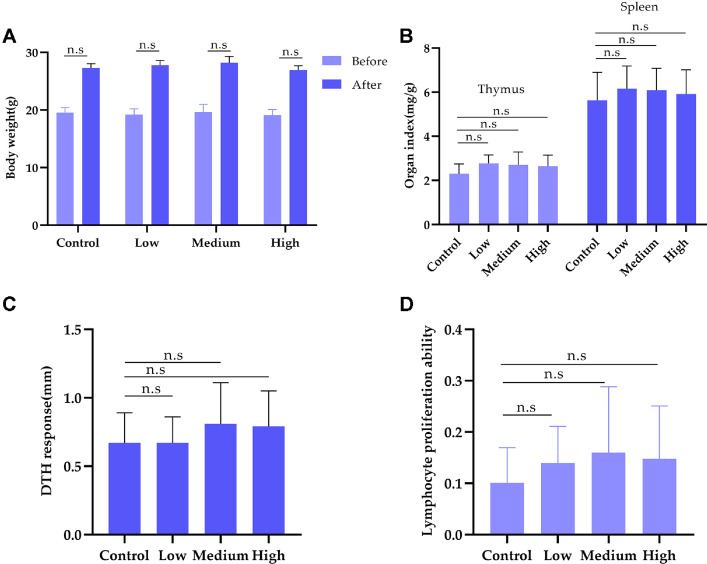
Effects of *LRa05* on general physiology and T cell immune response in mice. Effect of *LRa05* on body weight (**A**) and immune organ index (**B**) in mice (*n* = 12). (**C**) Effect of *LRa05* on DTH reaction (n =12). (**D**) Effects of *LRa05* on the proliferation of spleen lymphocytes in mice, as well as control group. Data are presented as mean values ± SD (*n* = 12 independent samples). Statistical significance was calculated by one-way analysis of variance (ANOVA). n.s means nonsignificance.

**Effect of *LRa05* on murine body weight and immune organ index.** In a 30-day experimental regimen, the mice were orally administered varying concentrations of *L. rhamnosus* *LRa05* solution via gavage. A comprehensive analysis of the somatic weight trajectories across all experimental factions was conducted. The findings delineated a non-significant yet discernible elevation in the terminal body weight of the experimental murine groups relative to the negative control cohort (Fig. 1A and Table S2). This observation intimates that the ingestion of distinct dosages of *LRa05* exerts a modest influence on murine weight gain.

Furthermore, the thymus and spleen, pivotal immunological viscera, are indices of the immune system's functionality, with their dimensions being indicative of immunomodulatory effects [34, 35]. The graphical representation in Figure 1B and Table S3 illustrates that *LRa05* induced a variable yet non-significant augmentation in the immune organ indices of the thymus and spleen when contrasted with the control group. Hence, it is tentatively surmised that the consumption of *L. rhamnosus* *LRa05* may confer a marginal enhancement in the immunocompetence of mice.

This interpretation is predicated on the premise that subtle modulations in immune organ indices could signify underlying immunoenhancing properties, thereby warranting further investigation into the immunological ramifications of *LRa05* supplementation.

### Effect of *LRa05* on Delayed Type Hypersensitivity Reaction (DTH) in Mice

The delayed-type hypersensitivity (DTH) response was employed as a metric to assess the efficacy of T-lymphocyte antigen recognition following re-challenge with the antigenic stimulus, specifically the sheep red blood cells (SRBC). SRBCs are known to activate T lymphocytes, prompting their proliferation into sensitized lymphocytes. Upon subsequent SRBC exposure, an inflammatory response ensues at the site of antigenic incursion, characterized by local edema. The magnitude of this swelling is posited to be indicative of the proliferative capacity of murine lymphocytes [36].

As illustrated in Figure 1C and Table S4, a comparative analysis of the pedal edema among the experimental groups receiving varying doses of the treatment and the control group revealed no statistically significant divergence (*P* > 0.05). This finding suggests that under the parameters of the study, the DTH response, as measured by toe swelling, was not substantially modulated by the treatment regimen, thereby indicating no significant alteration in the T-lymphocyte-mediated immune response to SRBC challenge.

### Effect of *LRa05* on ConA-Induced Mouse Lymphocyte Transformation

To elucidate the impact of *L. rhamnosus* *LRa05* on cellular adaptive immunity, a suspension of splenic lymphocytes was exposed to a gradient of *LRa05* concentrations in an environment supplemented with Concanavalin A (ConA). ConA, a known mitogen, is capable of inducing the clonal expansion and differentiation of naive T cells into effector and memory T cells [37].

The experimental outcomes demonstrated a discernible, yet statistically non-significant, augmentation in the proliferative capacity of splenic lymphocytes among the experimental groups when juxtaposed with the control group (as depicted in Fig. 1D and Table S5, *P* > 0.05). It is reported that *LRa05* could improve the production of specific cytokines, such as TNF-α, IL-10, and IL-1β, maintain Th1/Th2 balance. In the mean time, inflammatory factors can also promote the proliferation and differentiation of immune cells, so as to enhance the immune ability of the body [27]. Hence, despite the absence of statistical significance, this phenomenon could interpret a potential enhancement in the transformation and activation of splenic lymphocytes in response to the presence of *LRa05*, meriting further exploration into its immunomodulatory potential. It is worth noting that the interpretation of this trend should be tempered by the lack of statistical significance and requires confirmation in adequately powered studies.

### Effect of *LRa05* on the Number of Antibody Generating Cells in Mice

In the hemolytic plaque forming cell (PFC) assay, an intraperitoneal inoculation of sheep red blood cells (SRBCs) was administered, followed by an integration with the splenocyte suspension of mice and the subsequent introduction of guinea pig serum complement. This complement-mediated process facilitates the dissolution of SRBCs in the vicinity of antibody-secreting splenocytes, culminating in the formation of observable hemolytic plaques. The quantification of these plaques serves as an index of the frequency of antibody-producing cells.

**Fig. 2:**
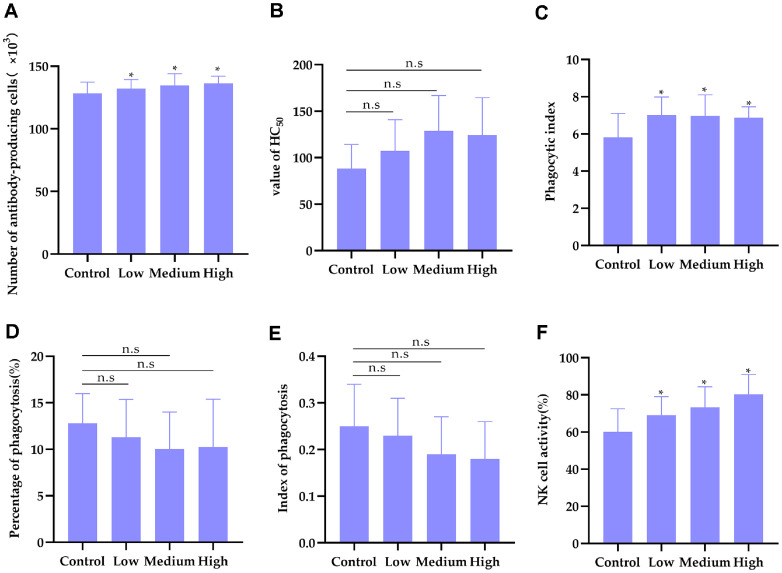
Effects of *LRa05* on humoral immune function, the phagocytic function of monocyte-macrophages and the activity of NK cells in mice. (**A**) Effect of *LRa05* on the number of antibody-generated cells in mice (*n* = 12). (**B**) Effect of *LRa05* on Half Hemolytic Value (HC_50_) of Serum Hemolysin in mice (*n* = 12). (**C**) Effect of *LRa05* on carbon clearance test (*n* = 12). Effect of *LRa05* on the ability of percentage of phagocytosis (**D**) and mouse macrophages to phagocytose index (**E**) (*n* = 12). (**F**) Effects of *LRa05* on NK cell activity in mice. NK cell activity was determined using LDH release assay (*n* = 12). Data are presented as mean values ± SD (*n* = 12 independent samples). Statistical significance was calculated by one-way analysis of variance (ANOVA). *p < 0.05 vs Control; n.s means non-significance.

The experimental data revealed a statistically significant elevation in the count of hemolytic plaques among the experimental groups relative to the control group (Fig. 2A and Table S6), with the distinction being markedly pronounced (*P* < 0.05 for the low and medium doses, *P* < 0.01 for the high dose). These findings underscore the potential of *L. rhamnosus* *LRa05* to augment the humoral immune response in mice, thereby promoting a significant increment in the population of cells engaged in antibody production.

This inference is drawn from the premise that an enhanced PFC response is indicative of an amplified humoral immune function, which could be attributed to the immunostimulatory properties of *LRa05*. Consequently, these results suggest that *LRa05* may serve as a promising candidate for bolstering the humoral arm of the immune system.

### Effect of *LRa05* on Half Cell Hemolysis Value (HC_50_) in Mice

The quantitative assessment of serum hemolytic activity, as reflected by the HC_50_ values, was conducted to evaluate the humoral immune response among experimental groups relative to a negative control cohort. Our findings indicate a modest yet statistically non-significant escalation in HC_50_ values across the three experimental groups administered with *L. rhamnosus* *LRa05*, with respective enhancement ratios of 21.14%, 45.45%, and 40.35% (Fig. 2B and Table S7).

This suggests a potential for *LRa05* to augment serum hemolysin production, a critical component of the immune system's defense arsenal. It is worth noting that the interpretation of this trend should be tempered by the lack of statistical significance and requires confirmation in adequately powered studies. However, upon rigorous statistical scrutiny, these variations did not achieve a threshold of statistical significance when contrasted with the control group (*P* > 0.05), implying that the impact of *LRa05* on humoral immune modulation in this study's design and parameters does not markedly deviate from the baseline established by the control group. These results necessitate further exploration to dissect the subtleties of *LRa05*'s influence on serum hemolysin dynamics and its overall immunoenhancing profile.

### Effect of *LRa05* on Carbon Clearance Ability of Mouse Mononuclear Macrophages

The functionality of the reticuloendothelial system was evaluated through the kinetic analysis of an inert marker, Indian ink, a standard procedure for assessing the system's activity. Within this immunological context, macrophages serve as the cornerstone of the defense mechanism, initiating the cascade that leads to T cell activation [38]. Phagocytosis, a fundamental process in immune defense, was triggered upon contact with Indian ink, with macrophages tasked with the systematic clearance of the marker from the bloodstream in a time-dependent fashion.

The experimental data demonstrated a marked increase in the phagocytic index (PI) among the groups treated with *L. rhamnosus* *LRa05*, as compared to the untreated control group (Fig. 2C and Table S8). The observed differences were statistically significant (*P* < 0.05), suggesting a potential upregulation of the reticuloendothelial system. This upregulation may have led to an enhanced production of patrolling phagocytes, indicative of a heightened systemic immune surveillance and response to foreign substances.

The findings imply that *LRa05* could modulate the reticuloendothelial system, possibly augmenting the host's immunological capabilities. The functionality of the reticuloendothelial system was evaluated through the kinetic analysis of an inert marker, Indian ink, a standard procedure for assessing the system's activity. Within this immunological context, macrophages serve as the cornerstone of the defense mechanism, initiating the cascade that leads to T cell activation [38]. Phagocytosis, a fundamental process in immune defense, was triggered upon contact with Indian ink, with macrophages tasked with the systematic clearance of the marker from the bloodstream in a time-dependent fashion.

### Effect of *LRa05* on the Phagocytic Capacity of Murine Peritoneal Macrophages

Macrophages serve as pivotal immune cells, orchestrating the innate immune response and forming the vanguard of the body's immunological defenses. As such, the phagocytic capacity of murine peritoneal macrophages constitutes a critical metric for assessing the functionality of the innate immune system [39]. The experimental outcomes indicate that both the phagocytic rate and index of the peritoneal macrophages across all experimental groups were reduced in comparison to the control group (Fig. 2D and 2E, and Table S9). However, this reduction did not attain statistical significance (*P* > 0.05), implying that the ingestion of a specific dosage of *L. rhamnosus* LRa05 may not substantially alter the phagocytic activity of the reticuloendothelial system under the conditions of this study.

### Effect of *LRa05* on NK Cell Activity in Mice

Natural killer (NK) cells are pivotal in the immune system's defense against tumor and virally infected cells, exerting their effects through mechanisms intricately linked to antitumor, antiviral, and immune regulatory activities [40]. In this study, the YAC-1 cell line, derived from a murine lymphoma and known for its sensitivity to NK cell-mediated lysis, was utilized as the target cell population. Splenocytes from mice, serving as the source of NK cells, were employed as effector cells. The assessment of NK cell activity was based on the measurement of lactate dehydrogenase (LDH) levels, an established marker for cellular cytotoxicity. The data, as presented in Fig. 2F and Table S10, demonstrate a statistically significant enhancement in NK cell activity following treatment with varying doses of *L. rhamnosus* LRa05 (*P* < 0.05). This augmentation in NK cell activity suggests that *LRa05* has the potential to invigorate the immune function *in vivo*, specifically by bolstering the non-specific immune response in mice.

### The 90-Day Oral Toxicity Experiments

**Fig. 3:**
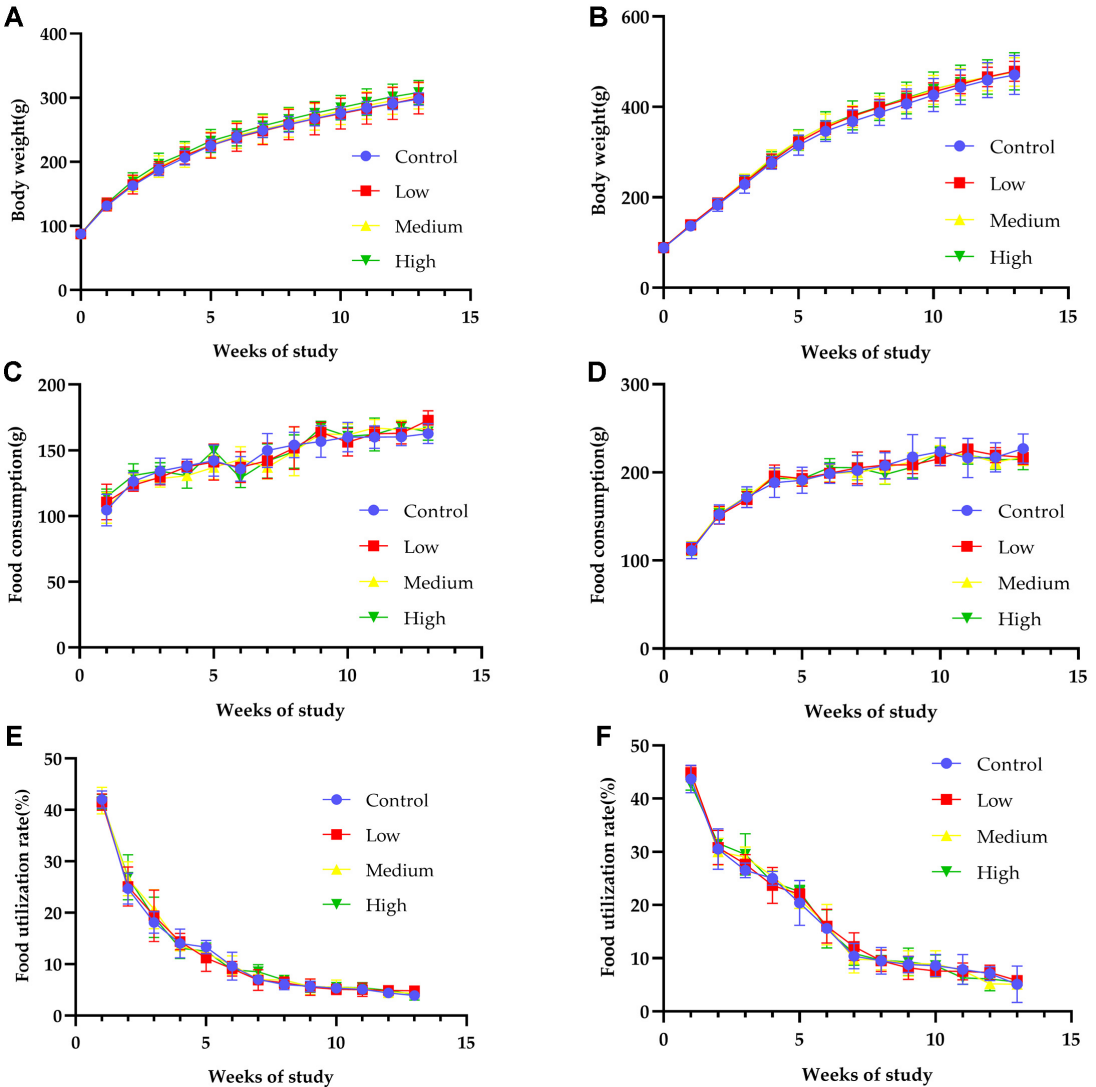
Average body weight, weekly food consumption, weekly food utilizations of rats treated with *LRa05* for 90 days. The average body weight of rats treated with *LRa05* for 90 days. (**A**) Females, (**B**) Males. Average weekly food consumption of rats treated with *LRa05* for 90 days. (**C**) Females, (**D**) Males. Average weekly food utilizations of rats treated with *LRa05* for 90 days. (**E**) Females, (**F**) Males. Data are presented as mean values ± SD (*n* = 10 independent samples). Statistical significance was calculated by one-way analysis of variance (ANOVA).

**Effect of *LRa05* food consumption, body weight and general condition index in rats.** In comparison to the control group, there were no abnormal manifestations or signs in any of the dosage groups administered *LRa05* throughout either the trial or recovery phases, and no deaths were observed. Additionally, as demonstrated in Fig. 3, there was no discernible difference between the *LRa05* dosage groups and the control group in terms of body weight (Fig. 3A and 3B, Table S11), food intake (Fig. 3C and 3D, Table S12), or food utilization rates (Fig. 3E and 3F, Table S13).

### Effects of *LRa05* on Hematological Parameters Index in Rats

As depicted in Table 2 and Table 3, during the intermediate and terminal phases of the study, no adverse impacts on the hematological indices were observed in either sex across the three dosage cohorts. The hematological parameters of the rats in the treatment groups did not exhibit statistically significant divergences from those of the control group, with all *p*-values exceeding the threshold of 0.05. Specifically, the administration of *LRa05* did not elicit significant alterations in the levels of hemoglobin (HGB), red blood cell count (RBC), white blood cell count (WBC), lymphocyte count (LYM), granulocyte count (GRA), mid-range cell count (MID), prothrombin time (PT), activated partial thromboplastin time (APTT), platelet count (PLT), and hematocrit (HCT) in the experimental subjects.

### Effects of *LRa05* on Clinical Biochemical Parameters Index in Rats

As illustrated in Table 4 and Table 5, upon the conclusion of the experiment, gamma-glutamyltransferase (GGT) levels in female rats from the medium and high-dose groups were found to be significantly reduced compared to the negative control group (*p* < 0.05). However, these values remained within the established normal reference range, and no dose-response correlation was discernible. Consequently, the observed variations in this parameter are not attributed to toxicological implications. Furthermore, no significant disparities were noted in the serum biochemical indicators among the remaining dosage groups and the negative control group, with all *p*-values surpassing the 0.05 threshold.

### Effects of *LRa05* on Organ Weight and Organ Weight Ratio in Rats

**Fig. 4:**
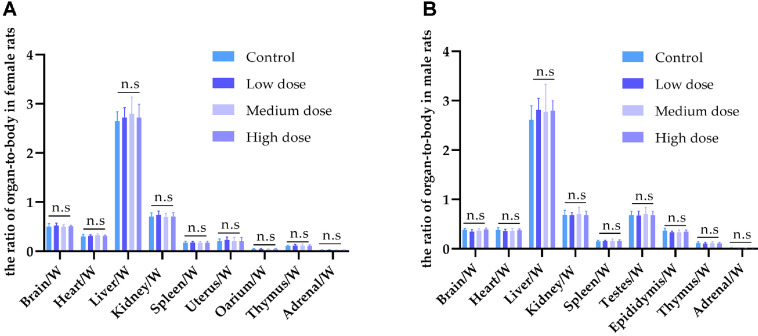
Effect of *LRa05* on the weight ratio of organ-to-body in rats. Effect of *LRa05* on the weight ratio of organ-tobody in female (**A**) and male (**B**) rats. Data are presented as mean values ± SD (*n* = 10 independent samples). Statistical significance was calculated by one-way analysis of variance (ANOVA). n.s means non-significance.

Compared with the control group, no significant alterations were observed in the relative organ weights or the organ-to-body weight ratios among the treatment groups for both genders (*p* > 0.05). The results are detailed in Figure 4A and 4B, Table S14 and Table S15.

### Effects of *LRa05* on Urinalysis Index in Rats

The experimental groups exhibited no significant deviations from the normative values. The urinary parameters, including pH, protein (PRO), glucose (Glu), blood (BLD), and specific gravity (SG), were all found to be within the acceptable physiological range, as delineated in Table 6 and Table 7.

### Effect of *LRa05* on Gross Anatomy and Histopathological Observation in Rats

A total of 80 rats, evenly divided by gender, were subjected to examination across the negative control group and the three experimental dosage groups. Following necropsy, a macroscopic examination was performed, which entailed a visual assessment of the color, size, and morphological integrity of vital organs. No significant anomalies were detected in the cardiac, hepatic, splenic, pulmonary, renal, cerebral, pituitary, thyroid, thymic, gastric, duodenal, pancreatic, jejunal, ileal, colonic, rectal, vesicular, lymphatic (mesenteric), adrenal, prostatic, testicular, epididymal, ovarian, and uterine tissues. These findings indicate the absence of macroscopic pathological changes attributable to the experimental intervention.

Upon histological evaluation of the myocardial tissue, the architecture was found to be intact, exhibiting crisply delineated muscle fibers, with no significant histopathological deviations observed in either the high-dose experimental group or the negative control group. This indicates a consistent myocardial integrity across both groups, suggesting that the treatment did not induce any adverse morphological changes.

Histological analysis of gastrointestinal tissues, including stomach, jejunum, and colon, from both high-dose and negative control groups, disclosed well-preserved mucosal, submucosal, muscular, and serosal layers without significant pathological findings; In the duodenum, an isolated instance of mucosal epithelial degeneration and necrosis was noted in one female from the high-dose group, which may be attributed to autolysis; In the ileum, the mucosal architecture was intact, featuring prominent capillaries and lymphoid follicles. Occasional mucosal epithelial degeneration and necrosis were observed in one male and one female from both high-dose and negative control groups, with autolysis considered a possible cause; Rectal tissues exhibited clear mucosal structures with interspersed goblet cells, and no abnormalities were detected in either group; Pancreatic lobules were uniformly structured with visible acini and islets; No significant changes were observed in liver lobules, with the exception of minimal inflammatory foci in one male from both high-dose and negative control groups, a single instance of hepatic stem cell vacuolar degeneration in one male from the negative control group, and minor inflammatory cell infiltration around hepatic blood vessels in one female from the negative control group. These findings suggest that the treatment did not induce substantial histological alterations in the majority of the examined tissues.

The pulmonary histology, encompassing bronchi, alveolar septa, and epithelial cells with predominantly clear structure. However, scattered inflammatory cell infiltrations were observed within bronchial, peribronchial, bronchiolar, submucosal, interstitial, and perivascular regions in 4 males and 4 females from the negative control group, as well as 4 males and 5 males from the high-dose group. In the negative control group, one male exhibited submucosal inflammatory proliferation in the tracheal submucosa with lymph node infiltration into the bronchial submucosa. Correspondingly, a high-dose group male presented with lymph node proliferation at the lung hilum, and a female displayed lymphocytic proliferation in the parabronchial area of the lung hilum. Alveolar cavities in 2 males and 1 female from the negative control group contained a modest accumulation of foamy macrophages. Additionally, one male in the negative control group showed signs of partial alveolar dilation with attenuated septal walls and focal alveolar congestion. A high-dose group female had a pulmonary apex inflammatory granuloma characterized by a neutrophilic abscess. These observations indicate localized pulmonary alterations in select individuals across both study groups.

The histological examination of renal tissues revealed intact renal capsules with normal glomerular and tubular structures. Interstitial inflammation was observed in two males from the negative control group and three males from the high-dose group. Degenerative changes in renal tubular epithelium were noted in one male from the negative control group and three males and one female from the high-dose group. A single male in the negative control group exhibited thinning of the renal medulla, suggestive of hydronephrosis, while a female displayed focal tubular degeneration with transparent tubules. Additionally, one male in the high-dose group had dilated tubules in the medullary region.

The urinary bladder showed a clear demarcation of the mucosal layer, muscular layer, and outer membrane with no significant abnormalities in either group. The cerebral gray and white matter were distinctly visible, with no vascular cuffing or glial nodules detected in the gray matter, and no significant differences were noted between the high-dose and control groups.

The thyroid gland displayed clear follicular structures filled with colloid, while one female in the negative control group showed residual thyroid parotid tissue. The adrenal cortex and medulla were well-delineated, with distinct zonal arrangements in the cortex, and no significant abnormalities were observed in either group. The pituitary gland showed an intact connective tissue capsule with a clear boundary between the neurohypophysis and adenohypophysis and abundant sinusoidal capillaries, with no significant findings in either group.

The thymus demonstrated clear lobular structures with intact connective tissue membranes and interlobular septa, filled with a dense lymphocytic network in the cortex and epithelioid cells in the medulla, with no significant abnormalities in either group. The spleen showed intact capsule and trabecular structures with clear demarcation between red and white pulp and normal splenic corpuscles, with no significant differences observed.

Lymph nodes exhibited intact trabecular structures with numerous lymphoid nodules and prominent germinal centers in the cortex, showing no significant abnormalities in either group. Ovaries displayed follicles and corpora lutea at various developmental stages with no significant abnormalities. The uterus showed no significant differences in cavity size or glandular structure.

Testes revealed clear seminiferous tubules with visible spermatogenic cells at different developmental stages. One male in the negative control group showed segmental atrophy of the seminiferous tubules with reduced sperm in the lumen. The epididymal duct showed clear structure with abundant mature sperm, while one male in the negative control group had reduced sperm production in the epididymal duct. The prostate gland showed clear internal structure with visible secretions, and one male in the high-dose group had focal prostatitis.

**Fig. 5:**
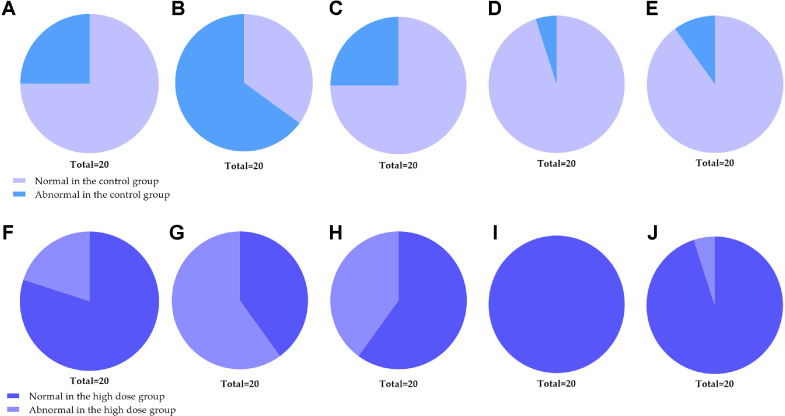
Proportion of normal and abnormal rats on organ-specific histopathological. Proportion of normal and abnormal rats in the control group (**A-E**) and high dose group (**F-J**) in the digestive system (**A, F**), the respiratory system (**B, G**), the urinary system (**C, H**), the endocrine system (**D, I**) and the reproductive system (**E, J**). Notes: the symptoms of abnormal rats in each group are detailed in the previous text.

Overall, the histological assessment indicated no significant pathological changes related to experimental factors in the hearts, liver, spleen, lungs, kidneys, brain, pituitary, thyroid, thymus, gastrointestinal tract, urinary bladder, lymph nodes, adrenal glands, prostate, testes, epididymis, ovaries, and uterus of both male and female mice in the high-dose group. Concurrently, no substantial effects on gross anatomy and histopathology were observed in the experimental rats. The results are detailed in Figure 5.

## Discussion

The present study elucidates the immunomodulatory properties of *L. rhamnosus* *LRa05*, underscoring its potential as a probiotic with health-promoting capabilities. Our findings are congruent with the existing literature, which posits that lactic acid bacteria can modulate gut microbiota equilibrium and bolster immune responses [41, 42]. Specifically, lactic acid bacteria have been reported to activate the intestinal mucosal immune system through diverse mechanisms [43, 44], safeguarding against infectious agents and exerting anti-mutagenic and anti-diabetic effects, as well as mitigating cardiovascular diseases [45, 46].

In our 30-day study involving mice administered varying doses of *LRa05*, no significant variations in body weight or organ-to-body weight ratios were observed, thereby substantiating the safety profile of *LRa05* [19]. Notably, a marked enhancement in the number of antibody-producing cells was detected across all dosage groups relative to the control, indicative of a robust humoral immune response. Furthermore, the carbon clearance assay revealed heightened phagocytic activity of monocytes and macrophages, and a pronounced upregulation in NK cell activity was noted. These outcomes, in accordance with the health food evaluation criteria outlined in the Methods for Functional Inspection and Evaluation of Health Food (2023 Edition), affirm the immune-strengthening function of *LRa05* [47, 48].

Concurrently, an oral toxicity study over a 90-day period with rats exposed to different concentrations of *LRa05* yielded no observable toxic manifestations or pathological alterations related to the treatment. The absence of detrimental effects on ocular health, body weight dynamics, dietary intake, hematological and biochemical parameters, urinalysis, organ weights, and organ-to-body weight ratios further corroborates the safety of *LRa05* consumption. Based on the intake levels of the high-dose group, the No Observed Adverse Effect Level (NOAEL) values were determined to be 9.27 g/kg BW for females and 8.6 g/kg BW for males, respectively.

While our study comprehensively assesses the impact of *LRa05* on murine immune function via a spectrum of indicators, including body weight, organ indices, cellular immune function (delayed type hypersensitivity, lymphocyte transformation), humoral immune function (antibody-producing cell count, hemolysis value), and immune cell activity (phagocytic capacity of monocytes and macrophages, NK cell activity), there are limitations. Notably, the scope of our investigation is confined to mice, and no experiments have been conducted on rats. Future research should encompass the analysis of white blood cell counts, lymphocyte subpopulations, and non-specific esterase staining in lymphocytes, as well as additional immunological markers such as ANAE+, PRV antibody titers, splenic histological assessments, and challenge test outcomes to enrich the empirical evidence supporting *LRa05*'s immunoenhancing effects[30]. Moreover, while the current study evaluates the safety of *LRa05*, it does not address reproductive toxicity or teratogenicity, which warrants further investigation.

Probiotics are live microorganisms that, when consumed in appropriate amounts, can benefit health, such as improving gut microbiota and boosting immunity [49]. However, the issue lies with individuals with immune deficiencies, whose immune systems are weaker and may not effectively control these external microorganisms like healthy people do [50]. Therefore, probiotics in immunodeficient individuals can pose serious risks, primarily including bacteremia or sepsis (due to live bacteria penetrating damaged intestinal barriers or contamination), the pathogenic potential of specific strains (such as *Lacticaseibacillus* and *Saccharomyces boulardii*), and inflammatory responses caused by overactivated immunity. Immunocompromised patients (such as those undergoing chemotherapy, organ transplant recipients, or HIV/AIDS patients) have weakened immune functions, making it difficult to control the spread of probiotics, significantly increasing their infection risk. Additionally, product quality issues (microbial contamination) and drug interactions (such as interfering with the metabolism of immunosuppressants) further exacerbate these risks. Clinically, it is recommended to strictly avoid using probiotics in patients with severe neutropenia or intestinal ischemia, and using them under professional medical supervision. Current guidelines (such as IDSA) only recommend providing probiotics to such populations during clinical trials, emphasizing the need for individualized “risk-benefit” assessments.

The safety of probiotics, in addition to their health benefits, must be determined to enable their commercial application. Our longitudinal analysis demonstrates that *L. rhamnosus* *LRa05* is safe. Moreover, the results of *in vitro* experiments reveal that *LRa05* has immunostimulatory properties. Furthermore, oral toxicity studies in rats show that *LRa05* has no pathogenicity or lethality. These findings demonstrate that *LRa05* is safe and lay the foundations for further studies on its efficacy in humans. In conclusion, our research underscores the immunopotentiating effects of *L. rhamnosus* *LRa05* and its safety as a dietary supplement. These attributes recommend *LRa05* as a promising candidate for integration into the food and health food industries. However, several avenues for future research are warranted. Future studies should explore the mechanisms underlying the immunoenhancing effects of *LRa05*, including its impact on gut microbiota composition and immune signaling pathways. Additionally, long-term clinical trials in humans are necessary to validate the safety and efficacy of *LRa05* in diverse populations. These studies will further elucidate the therapeutic potential of *LRa05* and support its broader application in functional foods and dietary supplements.

## Supplemental Materials

Supplementary data for this paper are available on-line only at http://jmb.or.kr.



## Figures and Tables

**Table 1:** Dose conversion in rats.

	Design dose/(g/kg BW/d)	Middle weight/g	Daily intake/g	The actual dose/(g/kg BW/d)
Female	0.333	226.5 ± 17.2	21.0 ± 0.6	2.38 ± 0.01
	0.667	228.7 ± 16.7	20.9 ± 0.4	4.68 ± 0.02
	1.333	233.3 ± 16.0	21.1 ± 0.1	9.50 ± 0.07
Male	0.333	330.5 ± 12.8	28.0 ± 0.3	2.17 ± 0.01
	0.667	332.4 ± 19.9	27.9 ± 0.4	4.30 ± 0.01
	1.333	332.4 ± 24.0	27.9 ± 0.2	8.60 ± 0.05

**Table 2:** Effect of *LRa05* on rat (intermediate) hematological parameters.

Project (unit)	Female		Male	
	Satellite control group	Satellite high dose group	Satellite control group	Satellite high dose group
HGB (g/L)	121.8 ± 3.8	131.6 ± 11.8	139.8 ± 5.0	127.8 ± 12.2
RBC (×10^12^/L)	5.86 ± 0.66	6.38 ± 0.72	6.44 ± 0.25	6.23 ± 0.45
WBC (×10^9^/L)	6.6 ± 1.8	5.6 ± 1.9	6.0 ± 1.8	6.9 ± 0.7
LYM (%)	73.4 ± 7.4	78.7 ± 5.6	84.5 ± 5.8	79.7 ± 2.6
GRA (%)	15.6 ± 6.1	13.7 ± 3.1	10.2 ± 3.9	12.9 ± 2.2
MID (%)	11.0 ± 2.5	7.6 ± 2.6	5.4 ± 2.1	7.4 ± 0.8
PT (s)	12.1 ± 0.8	12.0 ± 0.4	13.0 ± 0.6	12.9 ± 0.3
APTT (s)	14.4 ± 2.4	14.4 ± 0.8	16.7 ± 0.6	16.3 ± 0.7
PLT (×10^9^/L)	715.2 ± 97.7	763.2 ± 78.4	787.4 ± 113.7	698.2 ± 104.1
HCT (L/L)	0.34 ± 0.01	0.38 ± 0.06	0.37 ± 0.01	0.35 ± 0.03

**Table 3:** Effect of *LRa05* on rat (terminal) hematological parameters.

	Project (unit)	Control	Low dose	Medium dose	High dose
Female	HGB (g/L)	157.3 ± 4.9	156.4 ± 7.6	153.4 ± 13.4	158.4 ± 6.9
	RBC (×10^12^/L)	7.76 ± 0.33	7.75 ± 0.48	7.43 ± 0.87	7.80 ± 0.38
	WBC (×10^9^/L)	4.6 ± 1.3	3.9 ± 1.0	3.9 ± 0.9	4.4 ± 1.1
	LYM (%)	82.4 ± 6.8	78.6 ± 3.7	76.9 ± 3.8	80.6 ± 6.0
	GRA (%)	13.5 ± 5.8	16.7 ± 3.0	18.1 ± 4.1	15.3 ± 5.8
	MID (%)	4.1 ± 1.5	4.8 ± 1.4	5.0 ± 1.0	4.1 ± 0.9
	PT (s)	11.3 ± 0.7	11.1 ± 0.4	11.1 ± 0.5	11.3 ± 0.4
	APTT (s)	16.0 ± 1.5	16.6 ± 1.3	15.6 ± 1.3	16.8 ± 1.3
	PLT (×10^9^/L)	740.2 ± 59.3	683.0 ± 129.8	727.8 ± 181.7	727.0 ± 93.4
	HCT (L/L)	0.43 ± 0.02	0.43 ± 0.03	0.41 ± 0.04	0.42 ± 0.02
Male	HGB (g/L)	174.1 ± 6.1	174.2 ± 8.7	170.0 ± 10.3	171.4 ± 9.1
	RBC (×10^12^/L)	8.50 ± 0.49	8.66 ± 0.64	8.25 ± 0.80	8.52 ± 0.71
	WBC (×10^9^/L)	7.1 ± 1.4	7.0 ± 1.4	6.6 ± 1.6	6.6 ± 0.7
	LYM (%)	75.1 ± 7.4	79.3 ± 5.3	81.2 ± 3.2	80.6 ± 4.3
	GRA (%)	18.8 ± 6.6	15.5 ± 4.4	14.1 ± 2.3	14.5 ± 3.4
	MID (%)	6.1 ± 1.4	5.2 ± 1.3	4.7 ± 1.9	4.9 ± 1.4
	PT (s)	11.4 ± 0.7	11.9 ± 0.5	12.0 ± 0.7	11.7 ± 0.7
	APTT (s)	15.8 ± 1.0	15.8 ± 1.0	15.5 ± 0.7	16.1 ± 0.8
	PLT(×10^9^/L)	817.5 ± 131.3	802.0 ± 90.7	795.1 ± 129.0	779.0 ± 176.5
	HCT (L/L)	0.46 ± 0.02	0.46 ± 0.03	0.44 ± 0.03	0.45 ± 0.02

**Table 4:** Effect of *LRa05* on rat (intermediate) blood biochemical parameters.

Project (unit)	Female		Male	
	Satellite control group	Satellite high dose group	Satellite control group	Satellite high dose group
ALT (U/L)	31.9 ± 6.2	31.3 ± 4.5	39.3 ± 5.0	46.2 ± 8.5
AST (U/L)	119.6 ± 17.5	101.0 ± 24.0	139.7 ± 22.2	145.1 ± 26.9
TP (g/L)	65.2 ± 2.3	64.9 ± 1.8	59.9 ± 3.9	64.3 ± 7.9
Alb (g/L)	26.8 ± 1.4	25.9 ± 1.7	22.7 ± 1.4	22.9 ± 1.3
TC (mmol/L)	1.46 ± 0.13	1.55 ± 0.11	1.39 ± 0.16	1.43 ± 0.24
TG (mmol/L)	0.16 ± 0.01	0.19 ± 0.08	0.32 ± 0.07	0.28 ± 0.11
Glu (mmol/L)	4.96 ± 0.36	5.94 ± 0.92	5.82 ± 0.42	5.52 ± 1.00
BUN (mmol/L)	4.86 ± 0.41	6.07 ± 1.47	3.80 ± 0.70	4.14 ± 0.48
CR (mmol/L)	43.9 ± 4.0	47.4 ± 2.8	40.9 ± 5.0	41.2 ± 2.5
GGT (μ/L)	7.7 ± 0.6	7.3 ± 0.6	7.7 ± 1.4	7.8 ± 0.6
ALP (μ/L)	108.5 ± 10.2	118.6 ± 13.6	116.9 ± 11.8	103.7 ± 10.7
K (mmol/L)	4.54 ± 0.36	4.71 ± 0.47	5.00 ± 0.36	5.07 ± 0.36
Na (mmol/L)	143.2 ± 2.3	142.7 ± 0.6	144.4 ± 6.4	143.3 ± 0.8
Cl (mmol/L)	105.6 ± 3.8	105.0 ± 1.2	106.1 ± 5.4	105.3 ± 1.5

**Table 5:** Effect of *LRa05* on rat (terminal) blood biochemical parameters.

	Project (unit)	Control	Low dose	Medium dose	High dose
Female	ALT (U/L)	31.8 ± 5.5	32.5 ± 3.2	35.8 ± 3.8	32.4 ± 4.9
	AST (U/L)	105.5 ± 18.0	101.5 ± 13.4	112.0 ± 22.3	91.1 ± 17.6
	TP (g/L)	70.3 ± 2.0	67.4 ± 3.5	69.3 ± 3.8	68.5 ± 2.6
	Alb (g/L)	28.2 ± 1.7	28.2 ± 2.1	28.5 ± 2.0	27.3 ± 2.2
	TC (mmol/L)	1.47 ± 0.27	1.51 ± 0.34	1.58 ± 0.22	1.36 ± 0.23
	TG (mmol/L)	0.19 ± 0.04	0.18 ± 0.04	0.22 ± 0.06	0.16 ± 0.03
	Glu (mmol/L)	6.85 ± 1.22	5.62 ± 1.21	6.09 ± 1.04	5.58 ± 1.62
	BUN (mmol/L)	5.85 ± 1.10	6.31 ± .072	6.74 ± 1.23	5.95 ± 1.03
	CR (mmol/L)	53.6 ± 4.4	57.1 ± 4.6	56.7 ± 3.6	57.7 ± 3.4
	GGT (μ/L)	8.6 ± 0.6	7.9 ± 0.6	7.7 ± 0.7*	7.8 ± 0.8*
	ALP (μ/L)	58.5 ± 9.6	53.3 ± 17.1	62.7 ± 16.0	53.8 ± 17.2
	K (mmol/L)	4.43 ± 0.92	4.17 ± 0.59	4.16 ± 0.25	4.09 ± 0.44
	Na (mmol/L)	147.5 ± 2.7	148.2 ± 2.1	148.5 ± 2.3	148.6 ± 1.5
	Cl (mmol/L)	111.5 ± 1.9	109.3 ± 1.7	110.4 ± 2.7	111.8 ± 2.5
Male	ALT (U/L)	46.2 ± 8.0	49.2 ± 6.8	48.2 ± 7.0	49.7 ± 9.8
	AST (U/L)	134.8 ± 25.3	137.3 ± 32.0	113.6 ± 22.0	120.5 ± 16.8
	TP (g/L)	62.5 ± 4.1	64.7 ± 1.7	63.2 ± 2.0	62.7 ± 2.6
	Alb (g/L)	23.3 ± 1.4	24.3 ± 0.7	23.6 ± 1.0	23.6 ± 1.1
	TC (mmol/L)	1.43 ± 0.18	1.30 ± 0.21	1.35 ± 0.31	1.38 ± 0.33
	TG (mmol/L)	0.40 ± 0.16	0.36 ± 0.15	0.35 ± 0.19	0.47 ± 0.18
	Glu (mmol/L)	7.99 ± 0.50	7.82 ± 0.75	8.17 ± 0.41	8.01 ± 0.55
	BUN (mmol/L)	4.77 ± 0.96	5.02 ± 0.64	4.62 ± 0.65	4.32 ± 0.74
	CR (mmol/L)	45.2 ± 2.2	46.0 ± 3.2	46.9 ± 2.8	44.1 ± 2.1
	GGT (μ/L)	7.5 ± 2.4	6.1 ± 1.4	7.4 ± 1.7	6.6 ± 1.2
	ALP (μ/L)	102.7 ± 13.2	95.9 ± 14.2	99.9 ± 14.7	108.3 ± 6.8
	K (mmol/L)	5.20 ± 0.54	5.12 ± 0.33	4.87 ± 0.33	4.98 ± 0.35
	Na (mmol/L)	148.4 ± 3.3	148.9 ± 2.2	149.3 ± 0.9	149.3 ± 1.7
	Cl (mmol/L)	109.1 ± 1.5	109.1 ± 1.3	109.7 ± 1.6	109.8 ± 1.5

Each data point is presented as the mean ± standard deviation (SD). Statistical comparisons were made relative to the corresponding sex-matched satellite control group, *p < 0.05.

**Table 6:** Effect of *LRa05* on urine (female) indicators in rats.

	Project	Level	Female		Male	
			Satellite control group	Satellite high dose group	Satellite control group	Satellite high dose group
Female	Glu	-	10	10	10	10
		+-	0	0	0	0
	PRO	-	7	5	7	5
		+-	3	5	3	5
		|+	0	0	0	0
	BLD	-	10	10	10	10
		+-	0	0	0	0
	SG	1.005	5	5	7	7
		1.010	1	1	1	0
		1.015	4	4	2	3
		1.020	0	0	0	0
	pH	6.5	0	0	1	3
		7.0	5	6	4	5
		7.5	5	4	4	2
		8.0	0	0	1	0

**Table 7:** Effect of *LRa05* on urine (male) indicators in rats.

	Project	Level	Female		Male	
			Satellite control group	Satellite high dose group	Satellite control group	Satellite high dose group
Male	Glu	-	10	10	10	10
		+-	0	0	0	0
	PRO	-	2	2	1	2
		+-	8	7	7	8
		|+	0	1	2	0
	BLD	-	10	10	10	10
		+-	0	0	0	0
	SG	1.005	7	7	6	9
		1.010	2	2	2	0
		1.015	1	1	1	1
		1.020	0	0	1	0
	pH	6.0	0	0	1	1
		6.5	0	1	0	0
		7.0	7	2	6	2
		7.5	1	6	3	5
