# Supplementary material for: Longitudinal Analysis of the Immunostimulatory Properties and Safety Profile of Lacticaseibacillus rhamnosus LRa05 as a Dietary Supplement
Source: J Microbiol Biotechnol. 2025 Jul 14;35:e2502053. doi: 10.4014/jmb.2502.02053 (PMC12324998; doi:10.4014/jmb.2502.02053)
Supplement: Supplementary file 1 [file jmb-35-e2502053-supple.pdf]

J. Microbiol. Biotechnol. 2025. 35: e2502053
https://doi.org/10.4014/jmb.2502.02053

Supplementary Tables

Longitudinal Analysis of the Immunostimulatory Properties and Safety Profile of *Lacticaseibacillus rhamnosus LRa05* as a Dietary Supplement

Jiayi Wu [1], Li Zhou [1], Sitong He [1], Xuna Tang [2], Jue Wang [3,*] and Yanan Li [1,*]

[1]School of Food Science and Pharmaceutical Engineering, Nanjing Normal University, Nanjing 210023, P.R. China [2]Department of specialist clinic, Nanjing Stomatological Hospital, Medical School, Nanjing University 210008, P.R. China [3]National Institutes for Food and Drug Control, National Key Laboratory for Quality Control of Pharmaceutical Excipients, Beijing, China; Shanghai Medical Device and Cosmetics Evaluation and Verification Center, Shanghai, P.R. China

*Corresponding authors. E-mails: J.W.: myxwj2007@163.com;

Y.L.: liyanan@njnu.edu.cn

Table S1. Group of mice immune function experiments.

Groups	index
Immune Group One	Body weight and immune organ index,Delayed type hypersensitivity (DTH) reaction,Antibody generation cell detection,Half hemolytic value (HC50)
Immune Group Two	Carbon clearance assay
Immune Group Three	Ability of macrophages to engulf chicken erythrocytes
Immune Group Four	NK cell activity assay,Splenic lymphocyte proliferation

Table S2. Body weight changes of the mice in each experimental group.

Groups		Number of mice	initial weight/g	Final weight/g
Immune Group One	Control	12	19.55 ± 0.87	27.28 ± 0.78
	Low dose	12	19.22 ± 0.99	27.78 ± 0.82
	Medium dose	12	19.63 ± 1.33	28.20 ± 1.10
	High dose	12	19.09 ± 1.00	26.93 ± 0.75
Immune Group Two	Control	12	19.73 ± 0.84	27.30 ± 0.77
	Low dose	12	19.61 ± 0.78	27.32 ± 0.89
	Medium dose	12	19.84 ± 0.95	27.81 ± 0.87
	High dose	12	19.39 ± 0.69	27.02 ± 0.94

	Control	12	19.53 ± 0.72	27.24 ± 1.02
Immune Group	Low dose	12	19.41 ± 0.68	27.20 ± 1.21
Three	Medium dose	12	19.59 ± 0.84	27.88 ± 1.09
	High dose	12	19.30 ± 0.72	27.24 ± 0.79
	Control	12	19.53 ± 0.70	27.19 ± 0.82
Immune Group	Low dose	12	19.32 ± 0.58	27.03 ± 0.99
Four	Medium dose	12	19.48 ± 0.72	27.96 ± 0.75
	High dose	12	19.47 ± 0.79	27.38 ± 0.95

Table S3. Effect of *LRa05* on organ/weight ratio in mice.

**Groups**	**Number of mice**	**thymus index/(mg/g)**	**spleen index/(mg/g)**
Control	12	2.30 ± 0.45	5.63 ± 1.27
Low dose	12	2.78 ± 0.38	6.16 ± 1.03
Medium dose	12	2.70 ± 0.59	6.10 ± 0.98
High dose	12	2.64 ± 0.51	5.92 ± 1.10

Table S4. Effect of *LRa05* on delayed type hypersensitivity (DTH) in mice

**Groups**	**Number of mice**	**Swelling of toes(mm)**
Control	12	0.67 ± 0.22
Low dose	12	0.67 ± 0.19
Medium dose	12	0.81 ± 0.30
High dose	12	0.79 ± 0.26

Table S5. Effect of *LRa05* on mice lymphocyte transformation induced by ConA.

Groups	Number of mice	Lymphocyte proliferation ability
Control	12	0.101 ± 0.069
Low dose	12	0.139 ± 0.072
Medium dose	12	0.160 ± 0.128
High dose	12	0.148 ± 0.103

Table S6. Effect of *LRa05* on antibody-generating cell number.

Groups	Number of mice	Hemolytic plaque count($\times 10^3$)
Control	12	128.36 ± 8.93
Low dose	12	132.05 ± 7.34*
Medium dose	12	134.68 ± 9.35*
High dose	12	136.34 ± 5.76**

Table S7. Effect of *LRa05* on Half hemolytic value (HC_{50}).

Groups	Number of mice	HC_{50}
Control	12	88.47 ± 25.80
Low dose	12	107.17 ± 33.68
Medium dose	12	128.68 ± 38.17
High dose	12	124.17 ± 40.33

Table S8. Effect of *LRa05* on the carbon clearing capacity of mouse monocyte-macrophages.

Groups	Number of mice	phagocytic index
Control	12	5.82 ± 1.28
Low dose	12	7.01 ± 0.98*
Medium dose	12	6.98 ± 1.12*
High dose	12	6.87 ± 0.59*

Table S9. Effect of *LRa05* on the ability of mice macrophages to phagocytose chicken erythroid cells.

Groups	Number of mice	Percentage of Phagocytosis	Index of phagocytic
Control	12	12.78 ± 3.21	0.25 ± 0.09
Low dose	12	11.29 ± 4.08	0.23 ± 0.08
Medium dose	12	10.02 ± 3.99	0.19 ± 0.08
High dose	12	10.25 ± 5.14	0.18 ± 0.08

Table S10. Effect of *LRa05* on on NK cell activity in mice.

Groups	Number of mice	NK cell activity
Control	12	60.13 ± 12.37
Low dose	12	69.12 ± 9.88*
Medium dose	12	73.28 ± 11.06*
High dose	12	80.37 ± 10.58*

Table S11. Effect of *LRa05* on body weight in rats.

	Weeks	Control	Low dose	Medium dose	High dose
Females	0	87.6 ± 2.4	87.5 ± 2.0	87.3 ± 1.3	87.6 ± 3.5
	1	131.6 ± 7.6	133.4 ± 10.2	132.0 ± 7.6	135.3 ± 6.4
	2	162.8 ± 6.7	164.5 ± 14.6	165.7 ± 12.2	170.6 ± 12.5
	3	187.1 ± 8.7	189.9 ± 10.5	192.4 ± 16.6	196.4 ± 17.1
	4	206.5 ± 11.5	209.8 ± 13.4	210.3 ± 18.7	213.6 ± 18.0
	5	225.4 ± 9.8	225.6 ± 19.9	226.8 ± 19.6	232.4 ± 18.0
	6	238.7 ± 9.5	238.1 ± 21.8	241.0 ± 20.2	244.0 ± 19.4
	7	249.1 ± 11.1	248.2 ± 21.5	250.6 ± 22.6	256.0 ± 18.6
	8	258.4 ± 12.3	258.0 ± 23.8	260.9 ± 22.3	266.3 ± 18.8
	9	267.5 ± 11.2	267.0 ± 25.1	270.0 ± 20.7	275.7 ± 18.2
	10	276.0 ± 9.8	275.1 ± 24.0	279.0 ± 21.0	284.6 ± 19.1
	11	284.2 ± 10.0	283.2 ± 24.3	287.8 ± 21.2	293.3 ± 20.7
	12	291.3 ± 9.8	291.2 ± 25.1	295.5 ± 21.3	301.6 ± 19.8
	13	297.7 ± 9.9	299.4 ± 24.8	302.7 ± 20.5	308.3 ± 18.7
	Total weight gain	210.1 ± 10.4	211.9 ± 23.8	215.4 ± 20.4	220.7 ± 16.8
Males	0	88.0 ± 2.2	88.7 ± 2.0	88.9 ± 2.7	88.9 ± 4.7
	1	136.6 ± 5.5	139.4 ± 7.1	139.8 ± 6.2	136.9 ± 7.5

	2	183.2 ± 13.9	186.2 ± 12.0	186.1 ± 12.3	185.3 ± 10.4
	3	228.8 ± 19.5	233.1 ± 10.2	236.1 ± 15.1	236.5 ± 13.1
	4	276.1 ± 13.9	279.6 ± 15.9	286.2 ± 18.6	283.6 ± 17.3
	5	315.4 ± 22.0	322.1 ± 14.8	326.2 ± 21.1	326.9 ± 22.8
	6	346.4 ± 22.9	354.2 ± 14.5	358.9 ± 25.7	358.7 ± 30.4
	7	367.7 ± 25.7	379.5 ± 20.6	378.7 ± 24.2	381.2 ± 31.9
	8	387.4 ± 28.2	399.2 ± 17.6	398.8 ± 24.8	400.3 ± 30.3
	9	406.6 ± 33.3	416.6 ± 18.0	418.1 ± 29.4	419.6 ± 35.1
	10	426.0 ± 36.9	433.0 ± 20.1	437.8 ± 31.9	438.8 ± 38.7
	11	443.9 ± 38.9	449.9 ± 20.6	454.9 ± 30.3	453.8 ± 38.7
	12	459.1 ± 38.5	466.1 ± 21.6	465.9 ± 30.4	466.0 ± 38.2
	13	470.7 ± 43.1	478.7 ± 22.1	477.1 ± 31.4	478.8 ± 41.0
	Total weight gain	382.7 ± 43.0	389.9 ± 20.7	388.2 ± 30.7	389.8 ± 39.9

Table S12. Effect of *LRa05* on food intake in rats.

	**Weeks**	**Control**	**Low dose**	**Medium dose**	**High dose**
**Females**	1	104.6 ± 12.1	110.7 ± 13.5	106.3 ± 11.8	114.7 ± 5.8
	2	126.4 ± 6.5	123.5 ± 4.7	126.1 ± 6.0	130.9 ± 8.8
	3	134.2 ± 9.9	129.5 ± 5.9	128.5 ± 6.5	134.2 ± 6.4
	4	138.2 ± 5.0	137.5 ± 4.0	130.8 ± 3.6	130.7 ± 9.6

	5	142.2 ± 11.8	141.1 ± 13.7	137.1 ± 9.2	150.0 ± 4.6
	6	135.9 ± 9.3	137.3 ± 11.7	143.0 ± 9.8	128.9 ± 7.1
	7	149.9 ± 12.9	142.1 ± 13.6	137.0 ± 8.9	141.9 ± 13.0
	8	154.0 ± 9.8	151.8 ± 16.2	149.3 ± 18.5	149.0 ± 12.8
	9	156.8 ± 12.0	163.8 ± 7.5	162.8 ± 6.2	167.4 ± 4.7
	10	160.1 ± 11.2	156.3 ± 10.6	161.7 ± 8.3	160.7 ± 6.8
	11	160.0 ± 8.5	162.5 ± 5.1	167.0 ± 6.4	162.0 ± 12.5
	12	160.3 ± 6.8	163.0 ± 8.3	165.5 ± 7.2	168.1 ± 4.2
	13	162.8 ± 7.6	172.9 ± 7.1	166.9 ± 5.5	164.1 ± 6.3
	Total	1885.4 ± 28.1	1892.0 ± 51.8	1882.0 ± 32.8	1902.6 ± 13.4
**Males**	1	111.3 ± 9.4	113.0 ± 6.7	113.9 ± 7.8	112.4 ± 6.6
	2	152.4 ± 10.7	151.3 ± 10.0	153.7 ± 6.5	152.9 ± 6.5
	3	171.9 ± 11.7	169.1 ± 8.9	172.0 ± 7.9	172.9 ± 7.8
	4	188.4 ± 16.8	196.1 ± 12.2	196.7 ± 7.7	193.4 ± 6.8
	5	191.1 ± 14.8	193.2 ± 8.8	190.7 ± 5.0	192.6 ± 5.7
	6	198.7 ± 11.0	199.1 ± 10.6	199.0 ± 9.9	205.5 ± 10.1
	7	202.2 ± 17.0	205.0 ± 18.2	199.9 ± 9.0	205.3 ± 14.0
	8	207.7 ± 14.8	208.4 ± 15.9	205.5 ± 18.7	197.5 ± 11.0
	9	217.7 ± 25.3	208.5 ± 10.0	212.2 ± 10.1	206.2 ± 12.3
	10	223.7 ± 15.5	215.9 ± 8.2	221.7 ± 8.9	222.0 ± 6.8
	11	216.3 ± 22.4	225.5 ± 6.6	220.3 ± 9.5	219.7 ± 10.3

	12	217.1 ± 16.6	219.7 ± 8.5	211.3 ± 7.1	214.4 ± 10.9
	13	227.0 ± 16.6	217.1 ± 8.0	217.5 ± 9.8	215.0 ± 12.2
	Total	2525.5 ± 58.2	2521.9 ± 24.2	2514.4 ± 34.8	2509.8 ± 16.6

Table S13. Effect of *LRa05* on food utilization in rats.

	Weeks	Control	Low dose	Medium dose	High dose
**Females**	1	42.0 ± 1.7	41.5 ± 1.6	41.8 ± 2.6	41.6 ± 1.4
	2	24.7 ± 3.0	25.1 ± 3.8	26.6 ± 3.3	26.9 ± 4.4
	3	18.1 ± 2.1	19.4 ± 5.0	20.7 ± 3.8	19.1 ± 3.9
	4	14.0 ± 2.8	14.4 ± 1.6	13.6 ± 1.0	13.1 ± 2.0
	5	13.3 ± 1.3	11.2 ± 2.6	12.1 ± 1.1	12.5 ± 1.6
	6	9.6 ± 2.7	9.1 ± 1.4	9.8 ± 1.8	8.9 ± 0.9
	7	7.0 ± 0.9	6.9 ± 2.0	7.0 ± 1.0	8.5 ± 1.4
	8	6.0 ± 1.0	6.4 ± 1.1	6.9 ± 0.8	6.9 ± 0.5
	9	5.7 ± 1.0	5.5 ± 1.6	5.6 ± 1.5	5.6 ± 1.1
	10	5.3 ± 0.5	5.1 ± 1.0	5.5 ± 1.4	5.5 ± 0.8
	11	5.1 ± 0.8	5.0 ± 1.3	5.3 ± 0.9	5.4 ± 1.0
	12	4.4 ± 0.3	4.9 ± 0.5	4.6 ± 1.1	4.9 ± 0.4
	13	3.9 ± 0.2	4.8 ± 0.4	4.3 ± 0.9	4.1 ± 1.1
	Total	11.1 ± 0.5	11.2 ± 0.6	11.4 ± 0.3	11.6 ± 0.6
**Males**	1	43.7 ± 2.6	44.9 ± 1.3	44.7 ± 1.5	42.7 ± 1.1

2	30.5 ± 3.8	30.8 ± 3.2	30.0 ± 2.5	31.6 ± 0.9
3	26.5 ± 1.4	27.7 ± 1.8	29.0 ± 1.9	29.5 ± 3.9
4	25.0 ± 1.3	23.7 ± 3.4	25.4 ± 1.1	24.3 ± 0.6
5	20.4 ± 4.2	22.0 ± 1.0	21.0 ± 1.7	22.5 ± 1.2
6	15.6 ± 0.7	16.0 ± 3.2	16.3 ± 3.8	15.5 ± 3.6
7	10.4 ± 2.4	12.2 ± 2.6	9.8 ± 2.6	10.9 ± 2.2
8	9.5 ± 2.5	9.5 ± 2.0	9.7 ± 1.9	9.6 ± 0.6
9	8.8 ± 1.5	8.2 ± 2.2	9.0 ± 2.4	9.3 ± 2.6
10	8.6 ± 2.1	7.6 ± 1.0	8.9 ± 2.5	8.6 ± 2.0
11	7.9 ± 2.8	7.5 ± 1.6	7.7 ± 0.9	6.3 ± 1.2
12	7.1 ± 1.3	7.3 ± 1.4	5.2 ± 0.4	6.1 ± 2.2
13	5.1 ± 3.4	5.8 ± 0.6	5.1 ± 0.7	5.5 ± 0.6
Total	15.1 ± 1.4	15.5 ± 0.5	15.4 ± 0.7	15.5 ± 0.6

Table S14. Effect of *LRa05* on organ weight in rats.

	Project	Control	Low dose	Medium dose	High dose
**Females**	Brain	1.323 ± 0.158	1.391 ± 0.037	1.344 ± 0.049	1.426 ± 0.095
	Heart	0.813 ± 0.083	0.836 ± 0.094	0.890 ± 0.060	0.871 ± 0.079
	Liver	7.11 ± 0.61	7.30 ± 0.74	7.56 ± 0.61	7.54 ± 0.75
	Kidney	1.89 ± 0.17	2.00 ± 0.31	1.90 ± 0.21	1.97 ± 0.10
	Spleen	0.467 ± 0.086	0.480 ± 0.058	0.473 ± 0.068	0.485 ± 0.027
	Uterus	0.58 ± 0.12	0.62 ± 0.15	0.56 ± 0.17	0.59 ± 0.18
	Oarium	0.127 ± 0.030	0.131 ± 0.021	0.123 ± 0.023	0.137 ± 0.027

	Thymus	0.296 ± 0.037	0.306 ± 0.069	0.311 ± 0.084	0.308 ± 0.080
	Adrenal	0.068 ± 0.015	0.073 ± 0.012	0.067 ± 0.015	0.072 ± 0.008
	Fasting weight	267.6 ± 11.1	269.2 ± 22.7	271.4 ± 21.0	278.1 ± 18.9
**Males**	Brain	1.612 ± 0.106	1.549 ± 0.174	1.626 ± 0.152	1.676 ± 0.087
	Heart	1.640 ± 0.196	1.571 ± 0.096	1.556 ± 0.158	1.629 ± 0.112
	Liver	11.21 ± 0.94	12.34 ± 1.21	11.96 ± 2.08	12.22 ± 1.25
	Kidney	2.98 ± 0.31	3.01 ± 0.31	3.02 ± 0.52	2.97 ± 0.21
	Spleen	0.659 ± 0.059	0.683 ± 0.055	0.675 ± 0.171	0.695 ± 0.129
	Testes	2.906 ± 0.245	2.934 ± 0.428	2.998 ± 0.655	3.103 ± 0.388
	Epididymis	1.59 ± 0.17	1.46 ± 0.14	1.44 ± 0.15	1.53 ± 0.13
	Thymus	0.490 ± 0.107	0.468 ± 0.111	0.500 ± 0.123	0.489 ± 0.071
	Adrenal	0.085 ± 0.041	0.079 ± 0.008	0.078 ± 0.011	0.086 ± 0.006
	Fasting weight	431.9 ± 34.5	439.7 ± 24.2	435.7 ± 38.8	437.2 ± 38.6

Table S15. Effect of *LRa05* on the dirty body ratio in rats.

	**Project**	**Control**	**Low dose**	**Medium dose**	**High dose**
**Females**	Brain/W	0.50 ± 0.06	0.52 ± 0.05	0.50 ± 0.04	0.51 ± 0.02
	Heart/W	0.304 ± 0.034	0.311 ± 0.024	0.329 ± 0.030	0.313 ± 0.018
	Liver/W	2.65 ± 0.19	2.72 ± 0.20	2.80 ± 0.34	2.72 ± 0.27
	Kidney/W	0.71 ± 0.07	0.74 ± 0.08	0.70 ± 0.07	0.71 ± 0.08

	Spleen/W	0.174 ± 0.031	0.179 ± 0.024	0.175 ± 0.027	0.174 ± 0.030
	Uterus/W	0.21 ± 0.04	0.23 ± 0.06	0.21 ± 0.07	0.21 ± 0.07
	Oarium/W	0.047 ± 0.011	0.049 ± 0.007	0.045 ± 0.008	0.049 ± 0.010
	Thymus/W	0.111 ± 0.014	0.114 ± 0.028	0.116 ± 0.036	0.110 ± 0.022
	Adrena/Wl	0.025 ± 0.005	0.027 ± 0.004	0.025 ± 0.006	0.026 ± 0.004
	Brain/W	0.38 ± 0.03	0.35 ± 0.04	0.37 ± 0.03	0.39 ± 0.03
	Heart/W	0.381 ± 0.047	0.359 ± 0.035	0.361 ± 0.053	0.374 ± 0.028
	Liver/W	2.61 ± 0.29	2.81 ± 0.24	2.77 ± 0.56	2.80 ± 0.20
	Kidney/W	0.69 ± 0.09	0.68 ± 0.05	0.70 ± 0.14	0.68 ± 0.08
**Males**	Spleen/W	0.153 ± 0.018	0.156 ± 0.017	0.156 ± 0.042	0.159 ± 0.025
	Testes/W	0.68 ± 0.08	0.67 ± 0.09	0.70 ± 0.14	0.68 ± 0.08
	Epididymis/W	0.37 ± 0.05	0.33 ± 0.03	0.33 ± 0.05	0.35 ± 0.03
	Thymus/W	0.114 ± 0.027	0.106 ± 0.024	0.115 ± 0.029	0.113 ± 0.020
	Adrenal/W	0.020 ± 0.009	0.018 ± 0.002	0.018 ± 0.004	0.020 ± 0.002

Table S16. Number of normal and abnormal rats on organ-specific histopathological.

Groups		Normal	Abnormal
the digestive system	Control	15	5
	High dose	16	4
the respiratory system	Control	7	13
	High dose	8	12
the urinary system	Control	15	5

	High dose	12	8
the endocrine system	Control	19	1
	High dose	20	0
the reproductive system	Control	18	2
	High dose	19	1

File S1.*LRa05*-COA

LRa05-COA-WK-20231022.pdf
